# Exploring the Risk Factors and Possible Role of Calcium in Infective Endocarditis

**DOI:** 10.3390/diagnostics13142386

**Published:** 2023-07-17

**Authors:** Yen-Shou Kuo, Yuan-Ming Tsai

**Affiliations:** 1 Division of Thoracic Surgery, Department of Surgery, Tri-Service General Hospital, National Defense Medical Center, Taipei City 11490, Taiwan; geniuspipi@gmail.com; 2 Department of Cardiothoracic Surgery, University of Pittsburgh, Pittsburgh, PA 15213, USA

**Keywords:** risk factors, endocarditis, biomarkers, serum calcium

## Abstract

Infective endocarditis (IE) is a severe and potentially life-threatening infection that affects the endocardium, the inner lining of the heart chambers and valves. Although rare, it is a potentially fatal condition, with an incidence of 3–10 cases per 100,000 people per year in developed countries and a mortality rate of up to 30% within 30 days. Early identification and diagnosis are critical for improving outcomes. The diagnosis of IE typically involves a combination of biomarkers, blood cultures, and echocardiography. However, currently, there are no specific biomarkers for the early detection of IE. Given the lack of specific biomarkers for IE, serum calcium levels have been suggested to play a unique role in IE. There have been few articles on the correlation between serum calcium and IE, suggesting that patients with endocarditis and lower levels of serum calcium may have a poorer prognosis. Therefore, in this article, we focus on biomarkers of endocarditis and discuss the evidence showing serum calcium as a potential indicator of IE.

## 1. Introduction

Infective endocarditis (IE) has a global burden of 1.5–11.6 cases per 100,000 people and a high 5-year mortality rate of 40% [1]. IE is a serious infection of the heart endocardium, the inner lining of the heart chambers and valves. It is a rare, possibly secondary to central venous catheters, but potentially fatal condition, with an incidence of 3–10 cases per 100,000 people per year in developed countries and a mortality of up to 30% within 30 days [2]. IE is predominantly caused by bacterial infections, with the primary causes being degenerative valve disease, prosthetic valves, and *Staphylococcus* in high-income countries. In contrast, in low-income countries, a larger proportion of IE cases are attributed to Streptococci infection and rheumatic heart disease, with a higher prevalence observed among younger patients [3]. Other organisms that can cause IE include enterococci, fungi, and less commonly, viruses [4]. However, the data on risk factors and prognostic determinants of clinical outcomes in patients with IE are limited.

## 2. Pathophysiology of IE

The pathogenesis of IE involves a complex interplay between host factors, microbial virulence factors, and the presence of an abnormal or damaged heart valve or endothelium. The healthy endocardium possesses multiple defense mechanisms that help safeguard it against bacterial infections. First, the presence of endothelial cells lining the endocardium acts as a physical barrier that prevents bacteria from entering the bloodstream. Second, the endocardium mounts a natural immune response that involves the activation of several immune cells such as macrophages and neutrophils, which can engulf and destroy invading bacteria. Finally, the endocardium has the ability to repair itself after injury, which can help to prevent the formation of bacterial biofilms and limit bacterial colonization [5]. The infection usually begins at a site of endothelial damage or roughness, such as a congenital heart defect, prosthetic valve, or iatrogenic cause. The entry of bacteria into the bloodstream and their adhesion to the damaged endothelium are followed by the proliferation and formation of a biofilm. This biofilm shields the bacteria from host immune defenses and antibiotics, leading to persistent infection and potential complications such as embolization, heart failure, and systemic sepsis [6].

## 3. Risk Factors for IE

The most common risk factors for developing IE are degenerative valve disease, followed by a history of invasive procedures in the previous 60 days, congenital heart disease, intravenous drug use, and chronic intravenous access [2,7]. Damage to the endothelial surface can increase the likelihood of turbulent blood flow. This endothelial damage can lead to the formation of thrombotic vegetation, which develops when platelets and fibrin accumulate [8]. The likelihood of bacterial adhesion is greater in patients with a higher risk of developing bacterial infections, such as intravenous drug users, immunosuppressive patients, recent antibiotic usage or dental procedures [9], prosthetic heart valves, congenital heart disease, intravenous drug use, and a history of prior endocarditis [7].

## 4. Diagnosis of IE

The diagnosis of IE relies on a comprehensive evaluation of clinical features, blood cultures, echocardiography, and other imaging modalities. The modified Duke criteria are widely used to diagnose IE, which require the presence of either two major criteria, one major and three minor criteria, or five minor criteria [10]. The major criteria of the modified Duke criteria include positive blood cultures for a typical microorganism known to cause IE (such as *Streptococcus viridans* or *Staphylococcus aureus*), evidence of endocardial involvement, as demonstrated by echocardiography (either transthoracic or transesophageal), cardiac computed tomography (CT) or PET/CT, and evidence of endocardial involvement by intraoperative inspection. The minor criteria include a predisposing heart condition or intravenous drug use, fever of 38 °C or higher, vascular phenomena such as arterial emboli, septic pulmonary infarcts, or mycotic aneurysms, immunologic phenomena such as glomerulonephritis, Osler’s nodes, or Roth spots, or a positive blood culture not meeting major criteria or serological evidence of active infection with an organism consistent with IE, or abnormal metabolic activity as detected by PET/CT within 3 months of implantation of prosthetic materials (Table 1) [10]. The 2023 Duke Criteria propose significant changes, including new microbiology diagnostics (enzyme immunoassay for Bartonella species, polymerase chain reaction (PCR), amplicon/metagenomic sequencing), new imaging modalities (cardiac computed tomography, PET/CT), the inclusion of intraoperative inspection as a new Major Criteria, expansion of the list of “typical” microorganisms causing IE, removal of the requirements for timing and separate venipunctures for blood cultures, and clarification of additional predisposing conditions (transcatheter valve implants, endovascular cardiac implantable electronic devices, prior IE).

Although the modified Duke criteria are widely used in clinical practice and have been shown to have a high sensitivity and specificity for the diagnosis of IE, they should be used in conjunction with clinical judgment and other diagnostic tests, such as blood cultures and echocardiography [6]. Echocardiography is a non-invasive diagnostic tool that uses sound waves to create images of the heart. It plays a pivotal role in diagnosing infectious endocarditis because it can detect the presence of vegetation on heart valves, new valvular lesions, and other abnormalities in the structure and function of the heart [11].

Transthoracic echocardiography (TTE) is a non-invasive imaging modality that can detect IE in most patients. It is widely available, easy to perform, and relatively low risk. TTE can identify vegetations on heart valves and assess the sizes and locations of these vegetations. However, TTE has limitations in certain patient populations, such as those with prosthetic heart valves, those with a history of previous IE, and those with poorly visualized cardiac structures due to obesity or other factors [12]. In contrast, transesophageal echocardiography (TEE) is a more invasive imaging modality that is performed using a specialized probe passed through the esophagus to obtain images of the heart from a closer and more direct angle [13]. The main advantage of TEE over TTE is in diagnosing right-sided IE. In patients with implanted cardiac devices, vegetations are frequently located in the upper region of the right atrium, extending into the superior vena cava. In such situations, TEE can visualize lesions better than TTE [14].

Both TTE and TEE are useful to assess the size, mobility, and evolution of vegetation under antibiotic therapy. Additionally, it can identify potential complications, such as abscesses or emboli, which can be life-threatening if left untreated. TTE is the primary imaging modality used for initial evaluation in most patients with suspected IE, but TEE should be considered in certain situations, such as when TTE is inconclusive or when more detailed information is needed to guide treatment decisions.

## 5. Treatment of IE

The treatment of IE typically involves a combination of antibiotics and surgical intervention. Antibiotic therapy is individualized based on the identified causative organism and the severity of the infection. The proportions of microorganisms which induced the IE were *S. aureus* (26.6%), oral streptococci (18.7%), nonoral streptococci (17.5%), enterococci (10.5%), coagulase-negative staphylococci (9.7%), HACEK (*Haemophilus* spp., *Aggregatibacter actinomycetemcomitans*, *Cardiobacterium hominis*, *Eikenella corrodens*, *Kingella kingae*) microorganisms (1.2%), candida species (1.2%), and no microorganism identified (5.2%) [15]. Empirical antibiotics should be administered as soon as possible [16]. In addition, early surgical intervention for IE is associated with a lower risk of mortality and embolic events compared with medication alone [17]. Approximately 40–50% of patients with IE undergo surgical intervention [18]. The indications for surgery in IE can be classified into three categories: hemodynamic, infectious, and embolic indications. The objectives of surgery are to remove the source of infection and reconstruct the cardiac anatomy. Currently, surgical interventions for IE include valve repair and replacement [18].

To sum up, IE is a rare but potentially fatal condition that requires prompt diagnosis and treatment. It is important that clinicians should be aware of the risk factors, clinical features, and diagnostic criteria for IE, and should contemplate early empirical antibiotic treatment and surgical intervention in appropriate cases.

## 6. Biomarkers for the Diagnosis of IE

IE is a potentially life-threatening illness. The diagnosis of IE can pose challenges due to the potential non-specific nature of symptoms and negative blood samples in approximately 30% of cases. The delay period in diagnosing IE has been reported as 30 days [19]. Currently, the essential diagnostic procedure is echocardiography; TTE has a sensitivity of 61% and a specificity of 94%, whereas TEE has a sensitivity of 95% and a specificity of 100% for the diagnosis of IE [20]. Blood cultures are considered the gold standard for the diagnosis of IE as they can identify the causative organism. However, blood cultures may be negative in up to 30% of cases, particularly if antibiotics have been administered prior to blood collection [21]. By conducting three sets of blood cultures, it is possible to detect 96% of episodes of bloodstream infection [22]. Hoen et al. reported the combination of echocardiographic findings and positive blood cultures with a detection rate of 91% for the diagnosis of IE [23].

Biomarkers may play essential roles in the diagnosis and management of IE and are listed below:

C-reactive protein (CRP): CRP is an acute-phase protein that is elevated in response to inflammation. CRP facilitates the judgment of blood culture results and patient prognosis after the occurrence of infection [24]. Berk et al. reported a direct correlation between an acute myocardial infarction (MI) rise in CRP and postinfarction adverse events [25]. Elevated serum CRP levels were also associated with subsequent infarct size [26]. Several studies have demonstrated the usefulness of CRP in the diagnosis of IE, as it tends to be elevated in patients with IE [20,27]. Although CRP is a low-cost biomarker, it has drawbacks such as a lack of sensitivity and specificity and wide variation in the values between individuals, even in the absence of disease.

Procalcitonin (PCT): PCT is a precursor to the hormone calcitonin, and its levels rise in response to bacterial infection. Several studies have shown that PCT is useful in distinguishing IE from other conditions that can cause similar symptoms, such as non-infectious endocarditis, mitral valve prolapse, rheumatic fever, aortic stenosis, and myocarditis [28,29]. Cornelissen et al. reported the cut-off for PCT as >0.5 ng/mL, which demonstrated the highest accuracy in predicting an unfavorable outcome [30]. Higher values of PCT may also help to predict *S. aureus* infection [31]. However, there remains ongoing debate regarding the routine use of PCT as a biomarker of IE, as a suitable procalcitonin threshold for diagnosing or excluding IE has not yet been established [32].

Erythrocyte sedimentation rate (ESR): ESR can serve as an indicator of inflammation associated with infectious endocarditis. The ESR test measures the rate at which red blood cells settle to the bottom of a test tube [33]. In endocarditis, the inflammation caused by the infection can lead to elevated levels of fibrinogen and other acute-phase reactants, resulting in an increased ESR. Lamas et al. proposed modifications to the Duke criteria by including a high ESR as a minor criterion to enhance diagnostic sensitivity [34]. However, ESR is a non-specific marker of inflammation and may not be specific to endocarditis alone. Therefore, an elevated ESR may raise suspicion for infectious endocarditis, but a definitive diagnosis necessitates additional investigation and testing.

D-dimer: Plasma D-dimer is a protein fragment that is released into the bloodstream as a result of the dissolution of blood clots [35]. An increased plasma D-dimer level may indicate the presence of thromboembolic complications linked to endocarditis. However, it is important to note that elevated D-dimer levels are not specific to endocarditis and can be observed in various other conditions, such as deep vein thrombosis or disseminated intravascular coagulation [36]. Furthermore, an elevated plasma D-dimer level has been associated with thromboembolic complications and 6-month mortality in infectious endocarditis [37]. Nonetheless, further large-scale studies are necessary to validate the role of plasma D-dimer in the context of endocarditis.

N-terminal pro-B-type natriuretic peptide (NT-proBNP): NT-proBNP is a biomarker that is often used in the diagnosis and monitoring of cardiovascular disease, particularly heart failure. In patients with infectious endocarditis, NT-proBNP levels can be significantly elevated due to the cardiac stress caused by infection. Research has demonstrated that NT-proBNP levels can serve as a useful biomarker for diagnosing infectious endocarditis as well as monitoring treatment response and predicting outcomes [38,39]. However, elevated NT-proBNP levels can also be observed in other conditions, such as pulmonary embolism and acute respiratory distress syndrome [40,41].

Cystatin C: Cystatin C is a protein synthesized by all nucleated cells and is typically eliminated from the blood by the kidneys at a constant rate of breakdown [42]. Elevated levels of cystatin C in the blood can suggest impaired kidney function since the kidneys are responsible for filtering and eliminating cystatin C from the body [43]. Christian et al. reported that elevated levels of cystatin C at admission as well as after 2 weeks of treatment were independent prognostic markers for both 90-day and 5-year mortality in patients with IE. The inclusion of cystatin C into a multimarker composite risk scoring system has proven beneficial in identifying a high-risk group [44]. Nevertheless, to establish the diagnostic value of cystatin C in identifying infectious endocarditis, it would be beneficial to seek additional confirmation through large-scale cohort studies.

Troponins: Troponins are a group of biomarkers widely utilized in diagnosing and managing acute coronary syndromes, particularly MI. IE may cause cardiac damage and cardiac dysfunction, which can lead to cardiac injury and the release of troponins into the bloodstream. Multiple studies have suggested a correlation between elevated troponin levels in patients with infectious endocarditis and unfavorable outcomes, including higher mortality rates and prolonged hospital stays [45,46]. Furthermore, troponin levels can serve as a valuable indicator of treatment response in infectious endocarditis, as a decline in troponin levels may indicate improvement in cardiac function [47]. It is important to note, however, that elevated troponin levels can also be observed in other conditions such as pulmonary embolism, sepsis, and renal failure. Therefore, troponin levels should be interpreted in conjunction with other clinical information to establish an accurate diagnosis. 

S100A11: The Calcium-Binding Protein S100A11 is a member of the S100 family of calcium-binding proteins that play an important role in various physiological processes. Once bound to calcium ions, the conformations of the S100 proteins undergo changes, enabling them to bind to target proteins [48]. S100A11 has been found to be involved in regulating inflammation, cell proliferation, differentiation, and tumor development through binding to Toll-like receptor 4 (TLR4). Binding to TLR4 initiates a signaling cascade and regulates inflammation as well as other cellular processes through the activation of nuclear factor-κB (NF-κB) [49]. One study found that S100A11 was one of several biomarkers with promising potential for diagnosing bacterial IE [50]. In addition, S100A11 was correlated with the levels of lactate, a marker of inflammation, in patients with myositis [51]. Thuny et al. used the transcriptional signatures of blood samples to identify S100A11 as a potential diagnostic marker of IE [52]. However, additional research is needed to comprehensively understand the relationship between S100A11 and infectious endocarditis.

Aquaporins: Aquaporins (AQPs) are transmembrane channels that are involved in various physiological and pathological processes. AQP9 is expressed in multiple tissues, including the liver, spleen, and brain, and plays a role in the transport of glycerol and other small solutes [53]. However, there is no direct evidence suggesting the involvement of AQP9 in infectious endocarditis.

Cellular adhesion molecules: Cellular adhesion molecules (CAMs) are expressed on the surfaces of endothelial cells and leukocytes, playing a critical role in the adhesion and recruitment of leukocytes to sites of inflammation [54]. The formation of adhesion bonds between cells or between cells and the extracellular matrix is a crucial process in the pathogenesis of infectious endocarditis [55]. The study hypothesized that circulating levels of VCAM-1, E-selectin, and ICAM-1 may be useful markers for the increased expression of CAMs in infectious endocarditis [56]. Soderquist et al. reported that patients with endocarditis exhibited significantly higher concentrations of E-selectin and VCAM-1 in patients with *S. aureus* bacteremia and endocarditis [57]. Therefore, adhesion molecules, particularly CAMs, play a significant role in the pathogenesis of infectious endocarditis, highlighting their potential as diagnostic and therapeutic targets.

Interleukin-6: Interleukin-6 (IL-6) is a pro-inflammatory cytokine produced by various cells, including macrophages and endothelial cells, in response to infection or inflammation. In IE, the formation of vegetations on the heart valve triggers immune system activation, resulting in the release of pro-inflammatory cytokines such as IL-6, tumor necrosis factor-alpha (TNF-α), and interleukin-1 (IL-1) [58]. Elevated levels of IL-6 have been linked to the severity of endocarditis [59]. However, it is crucial to acknowledge that IL-6 is a non-specific marker of inflammation and may not be exclusive to endocarditis. For instance, elevated IL-6 levels have been linked to an increased risk of future MI in apparently healthy individuals [60]. Therefore, although elevated IL-6 levels may indicate the presence of endocarditis, additional diagnostic testing is required for a definitive diagnosis.

However, the gold standard for diagnosing IE still relies on a combination of clinical presentation, the results of blood culture, and echocardiography [50]. Echocardiography can detect valvular vegetations, abscesses, and other signs of cardiac involvement associated with IE [61]. The calcium deposition on the valve surface can be detected by echocardiography and is associated with an increased risk of embolic events [62]. Additionally, calcific aortic stenosis is a risk factor for the development of IE [63]. Therefore, the serum calcium level may be another biomarker to assist clinicians in the detection of IE.

## 7. Clinical Values of Serum Calcium as a Biomarker for Infectious Disease

Calcium ions (Ca^2+^) are implicated in many physiological processes, including neuronal transmission, muscle contraction, vascular constriction, hormone secretion, and immune response. At the cellular level, Ca^2+^ are crucial intracellular messengers that mediate gene transcription, cell proliferation, and apoptosis [64,65]. The serum Ca^2+^ level is a widely utilized clinical biomarker that offers several advantages in various medical conditions. In general, the serum Ca^2+^ level is used to assess the function of the parathyroid gland and to diagnose and monitor conditions related to Ca^2+^ metabolism. For instance, patients on chronic hemodialysis may be at an increased risk of developing IE due to increased Ca^2+^ deposition in cardiac valves [66]. Calcium is linked to several medical conditions, such as cardiovascular disease, osteoporosis, and cancer. Owing to its pleiotropic roles, Ca^2+^ imbalance can lead to multi-organ dysfunctions; therefore, the serum Ca^2+^ level may hold a unique value for the evaluation of IE and enable clinicians to utilize a low-cost and feasible parameter to consider the possibilities of inflammation and IE. However, there have only been a few articles addressing the issue of serum Ca^2+^ and infectious endocarditis. In the following section, we focus on related articles and discuss the available evidence for serum Ca^2+^ as a potential biomarker for IE.

### 7.1. Role of Calcium in the Pathogenesis of Infectious Endocarditis

Calcium homeostasis is critical to maintaining normal myocardial contraction and relaxation cycles. Calcium primarily enters the cell cytoplasm from the extracellular space via L-type Ca^2+^ channels, which trigger Ca^2+^-induced Ca^2+^ release from the sarcoplasmic reticulum via activation of the cardiac ryanodine receptor during myocyte contraction [67]. During the process of cardiomyopathy or myocardial dysfunction, fluctuations in Ca^2+^ levels may occur. In heart failure, an increase in cytosolic Ca^2+^ levels can be caused by excessive Ca^2+^ entry into the cytosol or reduced Ca^2+^ efflux from the cytosol [68]. An intracellular Ca^2+^ leak causes mitochondrial Ca^2+^ overload and dysfunction in post-ischemic heart failure [69,70]. Li et al. reported that serum Ca^2+^ presents a certain advantage in the early detection of sepsis in elderly patients and in assessing the severity of the disease [71]. The serum Ca^2+^ levels of elderly patients with sepsis were lower, indicating that the more severe the sepsis, the lower the serum Ca^2+^ levels. Sepsis patients with decreased serum Ca^2+^ tend to exhibit higher shock rates and mortality.

Cardiovascular morbidities are complex dysfunctions that involve an interplay between Ca^2+^ levels and inflammation in their development and progression. Celes et al. reported a significant increase in intracellular Ca^2+^ and calpain-1 levels in cardiomyocytes in a septic mouse model, suggesting a potential involvement of Ca^2+^ dysregulation in the development of cardiomyopathy or myocardial dysfunction associated with severe sepsis [72]. Inadequate delivery of oxygen and glucose to the brain, which receives 20% of the total cardiac output at rest, can cause dysfunction of the sodium-potassium pump, dysregulation of Ca^2+^, and cellular swelling. Furthermore, the lack of oxygen and the increase in intracellular Ca^2+^ concentrations can induce the production of pro-inflammatory cytokines. These findings highlight the significance of Ca^2+^ homeostasis and inflammation in the pathogenesis of cardiovascular diseases and suggest that targeting these pathways may hold therapeutic potential for managing these conditions [73]. 

The generation of reactive oxygen species induces mitochondrial injury and apoptosis [74]. The Calcium-Sensing Receptor (CaSR) is a G-protein-coupled receptor responsible for detecting fluctuations in extracellular Ca^2+^ levels and regulates Ca^2+^ homeostasis, inflammation, and cell apoptosis [75]. In some cases of endocarditis, bacteria can cause damage to heart valves, leading to the deposition of Ca^2+^ deposits. The presence of Ca^2+^ deposits can activate CaSR, which can lead to the production of pro-inflammatory cytokines and an increased risk of cardiac damage [76]. Therefore, modulating the activity of CaSR may have therapeutic potential for treating endocarditis. However, further research is necessary to comprehensively elucidate the relationship between CaSR and endocarditis.

### 7.2. Hypercalcemia and Infectious Endocarditis

Hypercalcemia is a well-known risk factor for pancreatitis [77], but only a few articles have addressed the correlation between hypercalcemia and infectious endocarditis. Hypercalcemia can manifest as a diverse range of symptoms, such as bone pain, neuropsychiatric disorders, constipation, nausea, nephrogenic diabetes insipidus, and kidney stones [78]. An et al. reported a 56-year-old woman with IE secondary to bacteremia related to untreated hyperparathyroidism-induced obstructive pyelonephritis. Untreated hyperparathyroidism can result in hypercalcemia and nephrolithiasis, which can increase the risk of obstructive pyelonephritis and potentially progress to bacteremia. If left untreated, bacteremia can spread to the heart causing endocarditis. Therefore, the serum Ca^2+^ level can serve as a crucial indicator of the risk of endocarditis, considering that untreated hyperparathyroidism can result in hypercalcemia, which may act as a contributing factor [78]. In immunocompromised patients, *Mycobacterium abscessus* infection can cause severe hypercalcemia and native valve endocarditis due to a peripherally inserted central catheter line [79,80]. The increased risk of endocarditis associated with *Mycobacterium abscessus* infection may be linked to the development of hypercalcemia. Hypercalcemia, in turn, can cause a range of cardiac abnormalities, including arrhythmias, heart failure, and valve calcification. These cardiac complications can increase the risk of developing endocarditis. However, further research is needed to fully understand the correlation between *Mycobacterium abscessus*, hypercalcemia, and endocarditis. Bosch reported that identical twins with cat scratch disease presented with hypercalcemia due to endogenous overproduction of active vitamin D. *Bartonella henselae* may cause hypercalcemia and endocarditis in immunocompetent patients [81,82]. 

Elevated serum Ca^2+^ levels can be caused by various conditions, such as primary hyperparathyroidism (PHPT), malignancy, vitamin D intoxication, and sepsis. PHPT is the most common cause of hypercalcemia and is characterized by the excessive secretion of parathyroid hormone (PTH) from one or more parathyroid glands. Sepsis, on the other hand, is a rare cause of hypercalcemia that may occur due to the increased production of cytokines, such as IL-1 and TNF-α. Measuring PTH levels can help distinguish between these two conditions. In PHPT, the PTH level is usually elevated or inappropriately normal despite the presence of hypercalcemia. In sepsis, however, the PTH level is typically low or suppressed. Additional laboratory tests that can assist in the differential diagnosis include measuring serum phosphate, chloride, 25-hydroxyvitamin D, 1,25-dihydroxyvitamin D, and calcium-to-creatinine clearance [83]. These laboratory tests, along with clinical findings and other diagnostic procedures, can assist in differentiating diseases as causes of hypercalcemia. Although there have been some reports on the relationship between hypercalcemia and endocarditis, the evidence is limited, and few studies have specifically addressed this issue.

### 7.3. Hypocalcemia and Infectious Endocarditis

In a case study by Rozsai et al., a 14-year-old girl with DiGeorge syndrome developed endocarditis and hypocalcemia. Based on their findings, the authors suggested that DiGeorge syndrome should be considered as a possible underlying condition in patients of all ages who present with endocarditis, particularly if hypocalcemia is present [84]. Chopra et al. reported that hypocalcemia can potentially induce subacute endocarditis due to resistance to the action of digitalis [85]. The above case reports revealed that hypocalcemia may have potential clinical value as a biomarker for endocarditis. In some cases, endocarditis can lead to hypocalcemia due to the formation of Ca^2+^ deposits in heart valves. The person may develop heart failure as a result of damaged heart valves, in which the heart cannot pump enough blood to meet the body’s requirements. Regarding the relationship between hypocalcemia and myocardial contractility, Ca^2+^ plays a crucial role in the contraction and relaxation of cardiac muscle cells. The process relies on Ca^2+^ from outside the cells entering and triggering the contraction since the Ca^2+^ stored inside the cells is insufficient to initiate this process. Inadequate levels of Ca^2+^ can impair the ability of the heart muscle to contract properly, potentially leading to decreased cardiac function [86]. According to the published articles, it was suggested that hypocalcemia may have contributed to the deterioration of cardiac function by impairing myocardial contractility and increasing the risk of arrhythmias and mortality [87,88,89,90] (Table 2). Consequently, low levels of Ca^2+^ in the blood may indicate the presence of endocarditis. Therefore, measuring Ca^2+^ levels in the blood can offer important diagnostic and prognostic information on patients with suspected endocarditis.

## 8. Conclusions

In conclusion, although there are few articles addressing the serum Ca^2+^ level as a biomarker for IE, we believe that patients with IE and lower levels of serum Ca^2+^ may have a poorer prognosis, indicating a potential relationship between endocarditis and serum Ca^2+^ levels. The integration of serum Ca^2+^ levels along with other biomarkers such as CRP, PCT, troponins, blood cultures, and echocardiography can significantly aid clinicians in the early diagnosis of IE and improve patient outcomes. However, the conclusions of this study still require confirmation through large-scale studies.

## Figures and Tables

**Table 1:** Summary of the 2023 Duke-ISCVID criteria for infective endocarditis **^a^**.

Criteria	Description
Major Criteria	*Microbiologic* (1) Positive blood culture for typical microorganisms ^**b**^ (2) Positive serology for Bartonella species (3) Positive molecular testing (PCR or sequencing) for typical microorganisms on blood or other sterile site *Imaging* (1) Evidence of endocardial involvement by echocardiography and/or cardiac computed tomography (CT) ^**c**^ (2) Evidence of endocardial involvement by PET/CT ^**d**^ *Surgical* Evidence of endocardial involvement by intraoperative inspection neither Major Imaging Criteria nor subsequent histologic or microbiologic confirmation
Minor Criteria	*Predisposition* Predisposing heart condition or intravenous drug use *Fever* Temperature > 38.0 degree Centigrade (100.4 degrees Fahrenheit) *Vascular phenomena* Clinical or radiological evidence of arterial emboli, septic pulmonary infarcts, cerebral or splenic abscess, mycotic aneurysm, intracranial hemorrhage, conjunctival hemorrhages, Janeway lesions, purulent purpura *Immunologic phenomena* Immune-complex glomerulonephritis, Osler nodes, Roth spots, positive rheumatoid factor *Microbiologic evidence* Positive blood culture but not meeting major criterion as noted previously or serologic evidence of active infection with organism consistent with IE. *Imaging* Abnormal metabolic activity as detected by PET/CT within 3 months of implantation of prosthetic valve, ascending aortic graft (with concomitant evidence of valve), intracardiac device leads or other prosthetic material *Physical examination* New valvular regurgitation identified on auscultation if echocardiography is not available.

ISCVID: International Society for Cardiovascular Infectious Diseases. **^a^** Definite IE is defined as meeting 2 major criteria, 1 major criterion with 3 minor criteria, or 5 minor criteria. Possible IE is defined as meeting 1 major criterion with 1 minor criterion or 3 minor criteria. **^b^** Typical microorganisms causing IE: Staphylococcus aureus OR—Streptococcus viridans group OR—Streptococcus bovis group OR—HACEK group (Haemophilus species, Aggregatibacter species, Cardiobacterium hominis, Eikenella corrodens, Kingella species) OR—Community-acquired Enterococcus faecalis without a primary focus OR—Coxiella burnetii OR—Tropheryma whipplei OR—In patients with prosthetic valves or other prosthetic material used for cardiac valve repair: Staphylococcus epidermidis and other coagulase-negative staphylococci; diphtheroids; Gram-negative bacilli; fungi; Legionella species. **^c^** Echocardiography and/or cardiac CT findings: vegetation, valvular/leaflet perforation, valvular/leaflet aneurysm, abscess, pseudoaneurysm OR—Intracardiac fistula OR—Significant new valvular regurgitation on echocardiography as compared to previous imaging or new partial dehiscence of prosthetic valve as compared to previous imaging. **^d^** PET/CT findings: abnormal metabolic activity involving a native or prosthetic valve, ascending aortic graft (with concomitant evidence of valve involvement), intracardiac device leads or other prosthetic material.

**Table 2:** Articles about hypocalcemia and infectious endocarditis.

Article Title	Authors	Discussion	Publication Year
Systemic immune-inflammation index predicts mortality in infective endocarditis	Agus et al.	A total of 133 patients (≥18 years) with diagnosis of definite IE were retrospectively assessed for ten years period. Systemic immune-inflammation index = platelet count × neutrophil count/lymphocyte count at admission In patients without in-hospital mortality (*n* = 103), the calcium level was found to be 8.6 ± 0.6. Conversely, in patients with in-hospital mortality (*n* = 30), the calcium level was 8.2 ± 0.7 (*p* value = 0.002). Hypocalcemia was independent predictors of in-hospital mortality (OR: 0.268, 95% CI: 0.083–0.862; *p* = 0.027).	2020 [88]
Characterization, epidemiological profile and risk factors for clinical outcome of infective endocarditis from a tertiary care centre in Turkey	Zencirkiran et al.	Total of 155 consecutive adult patients (18 years) admitted to single tertiary care hospital between 2009 and 2019 with definite infective endocarditis were retrospectively included in the study. The calcium level in patients without in-hospital mortality was 8.67 ± 0.69 (*n* = 120); The calcium level in patients with in-hospital mortality was 8.18 ± 0.72 (*n* = 35); *p* < 0.001 The hypocalcemia may be related to the in-hospital mortality.	2019 [89]
Hypocalcaemia in a patient with congenital heart disease	McCusker LA, Jenkins NP, Hancock JE.	Profound hypocalcaemia (corrected serum calcium 1.79 mmol/L) with relative parathyroid hormone deficiency (58 pg/mL) A case report with the 22q11 microdeletion syndrome represented with bacterial endocarditis and hypocalcemia.	2007 [90]
Infective endocarditis with hypocalcaemia and facial dymorphism in an adolescent girl	Rózsai et al.	Hypocalcemia was accompanied by hypomagnesaemia. Decreased glomerular filtration rate and microhematuria were considered to be the complication of endocarditis. The first case report of the diagnosis of DiGeorge syndrome after successfully treated infective endocarditis complicated with glomerulonephritis in an adolescent girl.	2007 [84]

## Data Availability

Data sharing not applicable; no new data were created or analyzed in this study. Data sharing is not applicable to this article.
